# Behavioral Changes During COVID-19 Confinement in France: A Web-Based Study

**DOI:** 10.3390/ijerph17228444

**Published:** 2020-11-14

**Authors:** Hélène Rossinot, Romain Fantin, Julien Venne

**Affiliations:** 1 Sharecare Europe, 8 rue de l’Hôtel de Ville, 92200 Neuilly-Sur-Seine, France; 2 Centro Centroamericano de Población, Universidad de Costa Rica, San José 11501, Costa Rica; romain.fantin@ucr.ac.cr; 3 Escuela de Salud Pública y Escuela de Medicina, Facultad de Medicina, Universidad de Costa Rica, San José 11501, Costa Rica; 4 UNU-MERIT (Maastricht Economic & Social Research Institute on Innovation & Technology), United Nations University, Maastricht University, 6211AX Maastricht, The Netherlands; julien.venne@sharecare.com; 5 Centre for Digital Health & Wellbeing (CDHW), Prasanna School of Public Health (PSPH), Manipal Academy of Higher Education, Manipal, Karnataka 576104, India

**Keywords:** COVID-19 1, behavioral change 2, public health 3, Sars-CoV-2 4, alcohol consumption 5

## Abstract

Background: A global pandemic due to COVID-19 emerged in November 2019 and hit France in early March 2020. It not only resulted in a loss of lives, but also in very strict confinement measures. The objective of this study was to understand what the determinants of the changes in participants’ behavior and mental state were during the confinement. Methods: An online survey was launched on 23 April 2020 and closed on 7 May 2020. The final sample included 1454 participants from 24 to 65 years old. Descriptive and multivariate analyses were then performed. Results: In total, 28.7% reported having a more balanced diet, against 17.1% with a less balanced diet, 22.7% of respondents reported an increased alcohol consumption, as opposed to only 12.2% declaring a decrease, and 11.2% of respondents increased their tobacco consumption, while 6.3% decreased it. In total, 50.6% of the participants reported being more depressed, stressed, or irritable since the beginning of the lockdown. Confinement had a negative effect on every behavior studied in this survey, except for nutrition. We also found that negative mental state changes were strongly associated with nutrition, sleep, physical activity and alcohol consumption changes.

## 1. Introduction

In November 2019, the novel severe acute respiratory syndrome coronavirus 2 (SARS-CoV-2), which causes the coronavirus disease 2019 (COVID-19), emerged in Wuhan, China. Since then, COVID-19 has rapidly spread around the world, leading to a pandemic that caused half of humanity to be in lockdown [1]. It thus not only resulted in medical complications and losses of lives, but has also led to the largest global economic impact on and transformation of daily life in modern times [2]. At the end (may 2020) of the survey here presented, there had been 145,746 confirmed cases and 28,596 confirmed deaths in France [3]. Confinement measures started on 17 March 2020 and were partially lifted on 11 May 2020. They consisted of a strong restriction on travel to only what was strictly necessary, such as food shopping, medication purchasing, care and work (when remote work was not possible) and outings close to home (limited individual sports activities, pets’ hygiene). Any breach in those rules could immediately be penalized by a fine, or prison in the case of recidivism.

As with a number of countries in the world, this was the first time that a lockdown had been implemented on the whole French territory in order to control a pandemic. Therefore, we have observed a lack of existing data concerning the impact of these preventive measures on the adult population. Thus, we have formulated three hypotheses underpinning our research. A better understanding of the potential negative effects of lockdown and their (external and individual) factors could lead us to propose more innovative public health policies and interventions to support the population in this difficult period. We also believe that this study’s findings could help to increase the level of preparedness of the citizens, the healthcare systems and the policymakers for new waves of the pandemic, or for the post-COVID-19 period, in the prevention of potential future epidemics.

Our first hypothesis was that the COVID-19-related preventive measures and restrictions (such as the confinement) had negative effects on the health and wellbeing of the adult population. Previous studies on the psychological impact of quarantine during other outbreaks, such as Ebola or SARS, found that they previously have resulted in deleterious mental health outcomes, including higher levels of depression [4], anxiety [5], irritability [5], post-traumatic stress symptoms [6], anger [7] and fear [8,9]. Limitations of social interactions and physical activity practices, both related to mental health, might partially explain these results [10]. Moreover, it has previously been shown that the deterioration of mental state could have consequences on the adoption of negative lifestyle habits, such as consumption of alcohol [11] and tobacco, sleep issues, poor nutrition [12] and poor physical activity. In France, COVIPREV, led by the national public health agency (Santé Publique France), and the study COCONEL [13] also showed a negative impact of the lockdown on mental health, and in particular on depression, and on health behaviors such as tobacco consumption, sleep issues or weight gain [14]. Hartley et al. [15] also conducted a study on the effects of the confinement of the French population on sleep. This is why we chose to focus on those behavior changes, which are particularly relevant in that context (alcohol and tobacco use, sleep, nutrition, physical activity).

Our second hypothesis was that the negative impact of lockdown and preventive measures could vary depending on individuals’ characteristics or external factors. On the former group of factors, we can mention here, as theoretical background, the concept of the social determinants of health (SDOH), defined by the WHO in 2008 as “the circumstances in which people are born, grow up, live, work and age, and the systems put in place to deal with illness. These circumstances are in turn shaped by a wider set of forces: economics, social policies, and politics.” Those social determinants of Health are complex and intertwined. The literature [16,17,18] has demonstrated the existing correlation between the effects of social, environmental and context-related factors and living conditions on the health and wellbeing of individuals, and the associated risk factors, via an influence on habits, behaviors, choices and lifestyles, as well as on resilience capacities. The second group of factors are related to the individual behaviors and lifestyles which impact the health and wellbeing of people [19]. A subsequent hypothesis we made was the potential intertwined effects that the two groups of factors could have together. The above-mentioned studies observed the negative impact and consequences of lockdown during previous epidemics, but they barely analyzed the groups of determinants. They also omit to analyze the relations between them. In order to verify our second hypothesis, we have analyzed in our model the impact of economic factors (with the evolution of employment at the moment of the survey and expectations in the near future), the neighborhood and the physical environment (housing or household conditions, access to outdoor leisure/sport area), the individual characteristics and behaviors around the physical activity, nutrition (diet), education (diploma), intrinsic COVID-19 risk factors, and community and social relationships.

In a nutshell, the objective of this study was to measure, characterize and understand the determinants of the negative impact of confinement on the health-related behaviors and the mental state of the French adult population, so as to contribute to the current expanding scientific knowledge about the pandemic’s dynamics and to inform the policy-making processes engaged in by governments, experts, health professionals and society as a whole.

## 2. Materials and Methods

### 2.1. Recruitment and Participants

In total, 1705 participants living in France and speaking French (as the questionnaire was written in French) participated in the online survey between 23 April 2020 and 7 May 2020. They were recruited through promoted social media posts. The final sample included 1454 participants after limiting participation to 24–65-year-olds, so as to have a sizeable sample (participation was quite weak between 18- and 24-year-olds and over 65-year-olds). Indeed, 115 participants were younger than 25 and 136 were older than 64 years old. The informed consent of participants was obtained. The survey was strictly anonymous and no re-identification was possible, therefore participants’ privacy was not at risk.

As the survey was not an interventional study, there was no need for ethical consent according to French laws [20]. The participants’ characteristics are described in Table 1 and Table 2.

### 2.2. Weighting

As the pandemic has not affected the whole country with the same intensity, based on the excess mortality observed between 1 March and 20 April 2020, in comparison with the same period in 2019, the INSEE created 5 groups of counties, ranked from highly affected (group 1) to not affected (group 5) [21].

For this study, Groups 4 and 5 will be unified because the distinction between very low and non-affected counties (with lower mortality in 2020 than in 2019) does not seem to make much sense because of the usual fluctuations in mortality from year to year at the county level.

The weighting of the sample was then based on three variables: gender, age, and the impact of the pandemic at the county level. The weights were therefore calculated so that the weighted sample corresponds to the distribution of the French population by age, gender, and impact of the pandemic.

### 2.3. Outcomes

Our survey asked respondents to evaluate the various components of their lifestyle and their situation in comparison with their status before the lockdown. The self-evaluation of the change during the lockdown of their diet (less balanced, no change, more balanced), sleep (worse quality, no change, better quality), physical activity (less, no change, more), alcohol consumption (more, no change, less), tobacco consumption (more, no change, less), tensions with the relatives (rise, no change), and the mental state indicator (0-to-3 score) were measured.

The mental health state indicator was created, as an additional score, using the addition of the answers to the question: “Since the beginning of the lockdown, are you feeling more… anxious? Depressed? Irritable?”. A score of 0 meant that the respondent had reported being more depressed, more stressed, and more irritable since the beginning of the lockdown. A score of 3 meant that the respondent did not notice any change.

Each outcome was ranked from the unhealthiest to the healthiest modality. The results of the outcomes are presented in Table 3. Please find in the Supplementary File S1 the questions asked to the participants in relation to the outcomes.

### 2.4. Independent Variables

Demographic (age, sex), socioeconomic (diploma), health (obesity, hypertension, other risk factors, ALD-status) variables, behaviors (smoke, alcohol consumption), the composition of the household (alone, with children), housing (overcrowding, outside access), the impact of the pandemic (around the respondent in her/his county based on INSEE data, on the participant’s and their relatives’ health, on their work, on their incomes), mental state indicator (0-to-3 score).

The ALD status is an “Affection de Longue Durée” (ALD), a major or long-term illness for which the State accepts responsibility for an individual’s health costs. It is defined in French law as a ‘condition requiring long-term care and particularly costly treatment’.

Three variables measuring concerns due to the situation were added as independent variables: concern for one’s health, for the health of one’s relatives, for one’s financial situation. These variables were ranked on a 1-to-10 scale, 10 being the highest concern, and were included as continuous variables in the model.

### 2.5. Model

Ordinal logistic regressions were used, except for tensions with relatives where a logistic regression was used. Observations were weighted to correspond to the French population by age, sex, and impact of the pandemic in the county. As all the outcomes were ordered from the unhealthiest to the healthiest modality, for each model, an odds-ratio superior to 1 was a protective factor and an odds-ratio inferior to 1 was a risk factor.

All the sample was used (*n* = 1454), except for two models.

In the alcohol consumption model, people who declared no alcohol consumption before and during the lockdown were excluded. For tobacco consumption, people who declared no tobacco consumption before and during the lockdown were excluded.

For tobacco consumption, people who reported no tobacco consumption before the lockdown were excluded. In this model, the mental state variable was divided into two (negative change or no change), due to sample size.

The selection of the independent variables was first made a priori. Health variables were only used in the diet and the physical activity models. Then we used a stepwise forward selection, with a *p*-value inferior to 5% for each model.

Table 4 presents the results of the multivariate analysis. The variables which were significantly associated with none of the outcome variables were excluded from Table 4.

## 3. Results

### 3.1. Descriptives

Participants’ characteristics are described in Table 1.

A majority of them were women (63.5%) and had studied 2 years or more after the Baccalauréat (84.3%). Most of them also had access to a private outside place during the confinement (80.0%). 41.3% of the participants were living in a territory where COVID-19 was very present.

A comparison of our sample with INSEE data shows that those who participated in the study had a level of education on average much higher than the French population [22] (Table 2). The variable was then recoded into three categories so that each category has a sufficient number of observations: Diploma less than Baccalauréat +2 (*n* = 229, 15.7%), Baccalauréat +2, Superior to Baccalauréat +2.

In total, 3.9% of respondents have received a COVID-19 diagnosis from their doctor, 0.8% have seen this result confirmed by a test and 12.1% think they were contaminated but were not diagnosed.

The majority of participants reported a decrease in mental state (50.6%), with 10.3% being at the same time more stressed, anxious, and depressed since the beginning of the confinement. Concerning sleep, physical activity, alcohol consumption and tobacco consumption, the percentage of participants who reported a negative change was higher than the percentage of participants who reported a positive change. Nutrition was the only outcome in which the percentage of participants who reported a positive change was superior to the percentage of participants who reported a negative change. Indeed, 28.7% reported having a more balanced diet, against 17.1% who reported having a less balanced diet since the beginning of the confinement.

Among the 418 participants who reported eating a more balanced diet, 70.8% had more time for cooking (*n* = 296), 41.4% ordered less take-out or used fewer delivery services (*n* = 173), and 17.2% snacked less during work (*n* = 72).

Among the 249 people who reported eating a less balanced diet, 58.6% snacked (*n* = 146), 43.8% considered eating more because they were stressed or sad (*n* = 109), and 26.5% were not motivated to cook (*n* = 66). It is interesting to note that 39.4% reported that they had more time to cook (*n* = 98).

Moreover, 22.7% of respondents reported an increased alcohol consumption, as opposed to only 12.2% reporting a decrease and 11.2% of respondents increased their tobacco consumption, while 6.3% decreased it (Table 3). Furthermore, 24.6% of the participants reported an increase in the tensions with their relatives, and in particular with their partner (14.3%). Finally, 50.6% of the participants reported being more depressed, stressed, or irritable since the beginning of the lockdown.

### 3.2. Multivariate Analysis

Table 4 shows the results of the ordinal logistic regression after the stepwise forward selection of the independent variables, for each outcome. Age was associated with the changes in diet, alcohol consumption, tensions with relatives, and mental state. An age range of 25–34 years old was a protective factor for both diet and alcohol consumption changes. An age range of 55–64 years old was also a protective factor for adverse diet changes. Increasing age was a strong protective factor against tensions with relatives and adverse mental state changes.

A negative change in mental state during the lockdown was a strong risk factor for negative changes in nutrition, sleep, physical activity and alcohol consumption. Concerning changes to mental state, our model shows a strong protective effect of age. Indeed, as age increases, so does the likelihood of not reporting stress, depression, or particular irritability since the onset of confinement. Similarly, men report fewer problems with stress, depression, and irritability than women. There is also a protective effect related to low educational attainment.

We observed that concerns related to the crisis itself have a strong impact on stress, depression, and irritability. This result remains similar when we exclude stress from this mental state score. This relationship is therefore not due to a conceptual redundancy between the measures of anxiety and mental health. Two measures of anxiety related to the crisis emerge significantly from our model: anxiety about one’s health, and anxiety about one’s finances. In both cases, worry was a strong risk factor in reporting any particular changes in mental state.

Being a man was a protective factor for alcohol consumption and negative mental state changes. A low education level was a protective factor for sleep and negative mental state changes. Alcohol consumption before the lockdown was a protective factor for negative physical activity changes. Health issues subjecting the participant to “severe form of COVID-19 risk factors” other than obesity and hypertension were a protective factor for preventing negative diet changes.

The impact of the lockdown on the participant’s work situation had different effects depending on the outcome. Teleworking was a protective factor against negative diet changes. Participants who had to stop working or lost their incomes reported more tensions with their relatives and it was a risk factor for negative alcohol consumption changes.

The concern for one’s health was a risk factor for negative mental state changes. The concern for one’s relatives’ health was a protective factor for alcohol consumption. Finally, the concern for one’s financial situation was a risk factor for alcohol consumption, negative mental state changes, and the rise of tensions.

The composition of the household and a change in mental state during the lockdown were strongly associated with various outcomes. Living alone was a strong risk factor for diet, sleep, physical activity, and tobacco consumption. It was a protective factor for tensions. Living with children was a risk factor for tensions and overcrowding for alcohol consumption.

### 3.3. Impact of Confinement on Mental State

Our results show that the older the respondents are, the less likely they are to report having symptoms of anxiety, depression, or irritability, and that younger people (25–34 years old) were more likely to present those symptoms. Those findings coincide with Huang and Zhao [23]’s preliminary publication, and also with Su et al.’s [24] results from the SARS epidemic. The deterioration of mental state had a negative effect on most behavioral indicators in our study (tobacco and alcohol consumption, physical activity and sleep).

Living alone was a strong risk factor for diet, sleep, physical activity, and tobacco consumption. This coincides with Bu et al.’s [25] preliminary findings stating that people living alone are at a higher risk of loneliness during the lockdown. In addition to quarantine, research into social isolation and loneliness indicates that these experiences are not only deleterious for mental health [26] but also have an impact on physical health, such that Nelson et al. [2] suggest that secondary effects on mortality due to COVID-19 may be caused by increases in social isolation and loneliness. Those findings concur with ours.

### 3.4. Impact of Confinement on Diet

In our survey, confinement had a positive effect on diet. However, these results apply only to people with a higher education level and probably depend on income.

This concurs with the results of Deschasaux-Tanguy et al. [27] which showed that improvements in the nutritional quality of diets were observed in a part of the studied population, including an increased AHEI-2010 score for diet quality, a decreased proportion of ultra-processed foods, increased intakes of fruit and vegetables, legumes or nuts, and decreased intakes of red meat or alcohol. These favorable behaviors mostly clustered participants with higher incomes and education levels, which matches our sample.

### 3.5. Impact of Confinement on Sleep

When asked if their sleep had been better or worse since the beginning of the confinement, respondents reported a decrease in sleep quality rather than an improvement, which is consistent with the findings of COCONEL [13] and COVIPREV [14], although in both of those studies the prevalence of sleep disorders was higher than in ours.

We found a significant difference between men and women, which concurs with Hartley et al.’s [15] study but runs counter to Huang and Zhao’s [23] preliminary publication. However, in Hartley et al.’s [15] findings, sleep-related factors were strongly associated with impaired sleep perception, with 90% of participants reporting shorter sleep, whereas in our survey the proportion of people for whom confinement resulted in an increase in sleep appears similar to the proportion of people for whom it resulted in a decrease, meaning that participants do not necessarily associate quality and quantity of sleep. During confinement, two things can decrease sleep pressure: a decrease in physical activity and an increase in anxiety that acts directly on the wakefulness systems [28].

### 3.6. Impact of Confinement on Tobacco Consumption

Our results show that confinement had a negative effect on tobacco consumption, as the proportion of participants who increased their consumption is double the proportion of those who decreased it. We found that living alone is a major risk factor of tobacco consumption during the confinement, which is not highlighted by COVIPREV [14], but also, participants who reported the negative effects of confinement on their mental state were less likely to have decreased their use, or more likely to have increased it, which this time coincides with COVIPREV [14].

### 3.7. Impact of Confinement on Physical Activity

Participants were more likely in our survey to report a decrease in their physical activity during confinement than to report an increase. Those results are consistent with the findings of Meyer et al [29]. We observed a strong association between physical activity and mental state. People who have reduced their physical activity are at greater risk of experiencing a deterioration in their mental health. On the other hand, there is no difference between those who did not change their physical activity habits and those who increased their activity.

### 3.8. Impact of Confinement on Alcohol Consumption

Our results concerning alcohol consumption were much more severe than COVIPREV’s [14] results, as 22.7% of our respondents reported an increased consumption, versus only 11% of their respondents. On the same trend, 12.2% of our respondents reported a decrease in their consumption, versus 24% of their respondents. The change in alcohol consumption since the start of confinement follows a U-shaped relationship with age in our survey. Indeed, 25–34-year-old respondents decreased their consumption much more often than the 35–54-year-olds. The questionnaire does not allow us to be completely positive about the cause of this result. Nevertheless, the 25–34-year-old participants who had reduced their consumption mentioned the fact that they only drink alcohol when they go out much more regularly (61.0%) than the other populations (32.3%) (*p* < 0.01). The 55–64-year-olds increased their drinking much less often than the 35–54-year-olds. These results are in opposition with COVIPREV’s [14] findings that alcohol consumption increased in the 50+ population. Finally, there are contradictory trends between concerns about COVID-19 and changes in alcohol consumption in our survey. People who were worried about their relatives were more likely to decrease their consumption, or not to increase it. On the opposite end, respondents who worried about their finances tended to increase their consumption or not to decrease it. This result may seem contradictory with the fact that not working seems to be a protective factor, all other things being equal. This shows that it is not the fact of not working per se that affects drinking, but the financial worry generated by the situation.

## 4. Discussion

Our results show that confinement and the associated restrictive measures had a negative effect on every individual behavior studied in this survey, except for nutrition. We also found that a negative change in mental state was strongly associated with adverse changes in nutrition, sleep, physical activity, and alcohol consumption. Furthermore, living alone was a strong risk factor for diet, sleep, physical activity, and tobacco consumption.

In our survey, the decrease in physical activity was also related to a worsening of mental state. Being a woman was a risk factor for concerns and age was a strong protective factor against negative mental state changes.

This survey was run in a short timeframe and has allowed the analysis of a large number of respondents (*n* = 1454). As there are very few data as yet on the behavioral changes of the French population during the confinement, our survey is important. Moreover, we analyzed various behaviors, whereas most of the few existing studies focused on sleep issues. Furthermore, our survey was completed during the confinement, which makes it more precise than a retrospective study, but not immediately after its beginning, allowing effects on behavior to show.

However, several limitations have to be mentioned, such as the online recruitment of the participants which implies a bias in the representativeness of the general population. We observed, for instance, that our sample included people mostly having a rather high educational profile or being located in specific geographic areas (in particular Paris and Great East regions) strongly affected by the crisis of COVID-19. Therefore, the respondents reported an astonishingly high level of compliance (99.3%) with preventive measures decided by the French government and local authorities. All territories were thus affected by confinement in the same way.

Moreover, our sample was composed of a majority of women. Nevertheless, the fact that women respond more often than men to surveys is a well-known phenomenon, and is therefore not specific to this survey. However, we used weighting to avoid this issue. Our analysis showed that the impact of these biases was limited.

The biggest limit of this study is that we do not know the participants’ behavior before the confinement. We can thus only get results on their behavior changes, but cannot precisely define their habits. This is especially true in the case of alcohol and tobacco consumption. We do not have quantitative measurements, but only the reported changes in consumption.

The proportion of respondents who think they have been contaminated is surprisingly high, since the estimates of the proportion of the French population that has been contaminated by the virus are between 4 and 8% (of which a large proportion is asymptomatic). Nevertheless, the COCONEL study [13] showed that 9% of French people thought they had been affected (1% had been diagnosed by a test or a doctor), which is not very far from our results, given that it was made in early April.

This survey was based on self-evaluation by the respondents for changes in the different parameters we analyzed. The conclusions should take into account the subjectivity of these evaluations. Nevertheless, this survey aimed to capture the respondents’ perception, their feelings, and their views on the impact of the pandemic and the associated lockdown situation. From this perspective, we assume that the answers are accurate.

It would be interesting to have information about the respondents’ consumption of media content, its frequency and intensity, its level of quality (disinformation), and whether it is via television, radio, newspapers or online content (social media), and to study whether this media consumption is linked or not to mental state and behavioral changes. We observed a clear higher sensibility of younger respondents (25–34 years old) in terms of the psychological impact of the crisis and lockdown situation. We could formulate the hypothesis that the limitation of social life related to the lockdown situation is an important factor leading to this result. Further research is needed to confirm this hypothesis.

It appears clearly that, except for the diet, all the analyzed health and wellbeing factors (sleep, physical activity, alcohol consumption and tobacco consumption) have changed negatively since the beginning of the crisis. We observe a strong impact on the living conditions (living alone is a risk factor) and on the mental state, itself related to the level of concerns (in particular concerns for one’s health or financial situation). However, in the different models we have developed, the impacts are predominately mutual, and our data do not allow us to determine the exact causality effect between these variables. We can legitimately conclude that these variables are correlated, but we cannot strictly define which variable is influencing the others. It seems sensible to understand all these variables as a system, setting the scene for a multi-variable interplay, with the variables influencing and retro-influencing each other. With this approach, we can consider the presence of vicious and virtuous circles. This hypothesis should as well be confirmed by further research.

Since the survey took place in April/May, the situation was completely new for the population and the effects were very strong. However, over time, we think that people might have developed strategies to cope with the effects of confinement (doing physical activity at home, socializing in other ways, etc.), so the “time” and “experience gained” effects could not be taken into account in this study, and future public health interventions will most likely have to adapt to this “learning curve” of individuals. People will most certainly behave differently in future confinements, and should be more able to maintain a healthier lifestyle despite the situation. Services have emerged and should continue to emerge to offer this maintenance of healthy behaviors.

## 5. Conclusions

The results of this study lead us to think that, given the multiplicity of determinants and their intertwined relationships, multi-factor interventions and a holistic approach are required to design the preventive and restrictive measures to control the pandemic. Thus, in order to limit their negative impact, decision-makers will have to balance their interventions across all factors (external and individual). This is illustrated by the recent debates around the prioritization of measures related to health security (strong limitation of social interactions) and economic and social needs (affecting greatly the mental state of the population). The prevention and public health policies should be accompanied by interventions supporting the population with coping strategies and resilience capacities.

Health and wellbeing programs might include specific interventions around physical activity, sleep management, and alcohol/tobacco consumption control/reduction. They should also provide people with advice on activities and best practices adapted to their specific context, but also increase the subjects’ motivation, embedding behavior change techniques leveraging key cognitive biases (social norms, messages framing, use of messengers, healthier default choices, ego, affects, etc.) potentially associated with incentives models (challenges and rewards). The interventions could also involve some coaching or counseling services around mental health, for instance about stress, or low spirits management and relationship management (to avoid tensions with relatives). Actions related to the improvement of the quality and validity of the information received by the population would also certainly benefit the global state of mental health.

Additionally, given that the intensity and the types of negative effects induced by lockdown or other preventive measures vary greatly depending on factors related to the living environment, as well as the lifestyle and choices of each individual, decision-makers should certainly integrate social determinants and social profiling methods to design their interventions. The population would benefit from more tailored-made measures depending on people’s location, living conditions, individual characteristics and preferences, etc. Therefore, this study shows us that the interventions deployed during and after the COVID-19 crisis would gain efficiency by using cost-effective tools, such as digital technologies [30], to deliver the above-mentioned components in a timely, personalized and remote manner.

Finally, the development of new frameworks for action in this context of crisis seems to be a priority. Indeed, decision-making processes require models and tools able to provide real-time tracking of a large spectrum of parameters (dashboards or digital twins of the living environment and of the population) to describe and explain the interactions between all these parameters, to deliver tailored interventions remotely to the population and other stakeholders (healthcare providers, employers, public services, education centers, etc.) and to simulate and test scenarios (via AI or machine learning) to anticipate the effects and improve the strategy ahead of the pandemic waves. Further scientific research and technological development will be required to enable this.

## Figures and Tables

**Table 1:** Participants’ characteristics.

Participants’ Characteristics	*n* * (%)
Age	
25–34	392 (27.0%)
35–44	427 (29.4%)
45–54	410 (28.2%)
55–64	225 (15.5%)
Gender	
Female	924 (63.5%)
Male	523 (36.0%)
Other	7 (0.5%)
Health issues putting the participant at risk of a severe form of COVID-19	
Obesity	103 (7.1%)
Hypertension	97 (6.7%)
Other risk factors	259 (17.8%)
ALD status	189 (13.0%)
At-risk behavior from the previous COVID pandemics	
Smoking (no detail on the amount)	403 (27.7%)
Drinking (no detail on the amount or frequency)	1076 (74.0%)
Diploma	
Less than 2 years of study after the Baccalauréat	229 (15.7%)
2 years of study after the Baccalauréat	218 (15.0%)
More than 2 years of study after the Baccalauréat	1007 (69.3%)
Impact of the crisis on the income	
Yes	498 (34.3%)
No	956 (65.7%)
Composition of the household during confinement	
Living alone	287 (19.7%)
Living with children	615 (42.3%)
Living with a vulnerable person	214 (14.7%)
Characteristics of the place of residence during confinement	
Overcrowded	81 (5.6%)
Access to a private outside place (garden, balcony, etc.)	1163 (80.0%)
Breakdown of the participants between groups based on the impact of COVID-19 on their territory of residence during confinement	
Group 1	601 (41.3%)
Group 2	373 (25.7%)
Group 3	168 (11.6%)
Group 4	312 (21.5%)
Mental state indicator	
0	150 (10.3%)
1	205 (14.1%)
2	381 (26.2%)
3	718 (49.4%)
Concern about COVID-19′s impact	Mean IC 95%
Concerns about one’s health	4.6 (4.5–4.7)
Concerns about one’s relatives	6.5 (6.4–6.6)
Concerns about one’s financial state	4.7 (4.5–4.8)
Total: *n* = 1454	

**Table 2:** Participants’ level of education.

Diploma	*n*	% (Sample)	% (Weighted)	% (INSEE *)
No diploma or certificate of general education	26	1.80%	2.20%	19.60%
CAP, BEP or equivalent	52	3.60%	3.80%	24.60%
Baccalauréat or equivalent	151	10.40%	10.60%	17.70%
Baccalauréat + 2	218	15.00%	16.70%	14.60%
>Baccalauréat + 2	1007	69.30%	66.80%	23.20%
Total: *n* = 1454				

* INSEE: Institut National de la Statistique et des Etudes Economiques.

**Table 3:** Outcomes.

Outcomes	*n* * (%)
Diet	
More balanced	418 (28.7%)
Unchanged	787 (54.1%)
Less balanced	249 (17.1%)
Tension with relatives	
Increased	357 (24.6%)
Unchanged	1097 (75.4%)
Sleep quality since the beginning of confinement	
Decreased	527 (36.2%)
Unchanged	737 (50.7%)
Increased	190 (13.1%)
Physical activity	
Decreased	506 (34.8%)
Started to practice physical activity during confinement	132 (9.1%)
Increased	248 (17.1%)
Stopped practicing physical activity during confinement	216 (14.9%)
Unchanged	352 (24.2%)
Alcohol consumption	
Decreased	178 (12.2%)
Unchanged	568 (39.1%)
Increased	330 (12.2%)
Do not drink	378 (22.7%)
Tobacco consumption	
Decreased	91 (6.3%)
Unchanged	149 (10.2%)
Increased	163 (11.2%)
Do not smoke	1051 (72.3%)
Mental state indicator	
0	150 (10.3%)
1	205 (14.1%)
2	381 (26.2%)
3	718 (49.4%)
Total: *n* = 1454	

**Table 4:** Regression results (*n* = 1454). The outcomes are ranked from the unhealthiest to the healthiest and the stepwise forward method was used. OR > 1 is a protective factor and OR < 1 is a risk factor.

Regression Results	Nutrition	Sleep	Physical Activity	Alcohol Consumption *	Tobacco Consumption **	No Tensions	Mental State
Age							
25–34 years	1	1	1	1	1	1	1
35–44 years	0.68 (0.50–0.93)	1.06 (0.77–1.45)	0.96 (0.71–1.31)	0.52 (0.37–0.74)	2.06 (1.18–3.60)	2.24 (1.53–3.27)	1.78 (1.34–2.36)
45–54 years	0.71 (0.52–0.96]	0.86 (0.63–1.17)	1.07 (0.80–1.43)	0.67 (0.47–0.940)	1.07 (0.62–1.85)	2.63 (1.81–3.83)	2.35 (1.76–3.14)
55–64 years	0.93 (0.69–1.25)	1.16 (0.86–1.57)	0.97 (0.72–1.29)	0.78 (0.55–1.10)	2.07 (1.18–3.62)	3.55 (2.39–5.27)	2.71 (2.02–3.64)
Gender							
Male	1	1	1	1	1	1	1
Female	0.97 (0.79–1.18)	0.95 (0.77–1.17)	1.17 (0.96–1.43)	0.71 (0.56–0.90)	0.96 (0.66–1.40)	0.98 (0.75–1.27)	0.77 (0.63–0.94)
ALD							
Yes			1.45 (1.10–1.92)				
No			1				
Other risk factors of severe COVID-19 form							
Yes	1.34 (1.03–1.74)						
Non	1						
Drank alcohol previously							
Yes			1.71 (1.36–2.15)				
Non			1				
Impact on work							
Telework	1			1		1	
Not working	0.85 (0.65–1.12)			1.71 (1.23–2.37)		1.24 (0.85–1.79)	
Works normally	0.58 (0.42–0.82)			1.19 (0.80–1.78)		0.74 (0.49–1.13)	
Others	0.62 (0.48–0.85)			1.25 (0.92–1.70)		0.72 (0.51–1.00)	
Household							
Living alone	0.64 (0.48–0.85)	0.75 (0.56–1.00)	0.73 (0.55–0.97)		0.44 (0.25–0.76)	4.08 (2.43–6.87)	
Living with children	0.86 (0.66–1.10)	1.14 (0.89–1.47)	0.81 (0.64–1.03)		0.69 (0.44–1.09)	0.44 (0.32–0.59)	
Others	1	1	1		1	1	
Overcrowded dwelling							
Yes				0.57 (0.32–0.99)			
No				1			
Mental state indicator							
0	0.39 (0.27–0.57)	0.12 (0.08–0.18)	0.47 (0.33–0.68)		0.65 (0.44–0.96)		
1	0.63 (0.46–0.86)	0.24 (0.17–0.32)	0.63 (0.47–0.86)				
2	0.85 (0.67–1.09)	0.39 (0.30–0.50)	1.01 (0.79–1.28)				
3 (No change)	1	1	1		1		
Diploma							
<Baccalauréat +2		1.55 (1.17–2.05)					1.33 (1.00–1.77)
Baccalauréat +2		1.28 (0.96–1.71)					0.83 (0.63–1.09)
>Baccalauréat +2		1					1
Concerns							
For one’s health							0.84 (0.81–0.88)
For one’s relatives				1.09 (1.04–1.15]			
For one’s finances				0.93 (0.89–0.97]		0.89 (0.85–0.93]	0.89 (0.86–0.92)

* *n* = 1076; ** *n* = 403.

## Supplementary Materials

The following are available online at https://www.mdpi.com/1660-4601/17/22/8444/s1. File S1: Enquete COVID avril 2020.
