# Supplementary material for: Behavioral Changes During COVID-19 Confinement in France: A Web-Based Study
Source: Int J Environ Res Public Health. 2020 Nov 14;17(22):8444. doi: 10.3390/ijerph17228444 (PMC7696923; doi:10.3390/ijerph17228444)
Supplement: Supplementary file 1 [file ijerph-17-08444-s001.pdf]

Enquete COVID avril 2020

Start of Block: Block 2

Display This Question:

IfDevice TypeIsMobile

Q90
Vous semblez répondre à ce questionnaire sur un téléphone portable : pour plus de confort, nous vous recommandons de vous connecter depuis un ordinateur.

Si vous préférez rester sur votre téléphone, vous pouvez essayer de le tourner pour trouver le mode d'affichage qui vous convient le mieux :)

Page Break

End of Block: Block 2

Start of Block: Questions eligibilite

Q87
Où résidez-vous ?

- ○ En France métropolitaine
- ○ En France outre-mer
- ○ Hors de France

Page Break

Display This Question:

If Où résidez-vous ? = En France métropolitaine

*

Q112 Veuillez rentrer les 2 chiffres du numéro de votre département :

____

Display This Question:

If Où résidez-vous ? = En France outre-mer

Q113 Dans quel territoire résidez-vous ?

- ○ Guadeloupe
- ○ Martinique
- ○ Guyane
- ○ Réunion
- ○ Mayotte
- ○ Autre

Page Break

Display This Question:

If Où résidez-vous ? != Hors de France

*

Q88 Quel est votre âge ?

____

Page Break

End of Block: Questions eligibilite

Start of Block: Default Question Block

Page Break

Q2 **Quel est votre sexe ?**

- ○ Femme
- ○ Homme
- ○ Je ne préfère pas le dire
- ○ Je préfère me définir autrement

Page Break

Q17 Quelle est votre situation professionnelle ?

- ○ Salarié(e)
- ○ Salarié(e) cadre
- ○ Fonctionnaire
- ○ Retraité(e)
- ○ Étudiant(e)
- ○ Indépendant(e)
- ○ Commerçant ou artisan
- ○ Au chômage
- ○ Autre

Page Break

Q91 Quel est le diplôme le plus élevé que vous ayez obtenu ?

- ◯ Aucun diplôme
- ◯ CEP (certificat d'étude primaires) ou diplôme étranger de même niveau
- ◯ Brevet des collèges, BEPC, brevet élémentaire ou diplôme étranger de même niveau
- ◯ CAP, BEP ou diplôme de ce niveau
- ◯ Baccalauréat général, technologique ou professionnel ou diplôme de ce niveau
- ◯ Diplôme de la santé et du travail social de niveau bac
- ◯ Capacité en droit, DAEU, ESEU
- ◯ Diplôme de niveau Bac+2 (DEUG, BTS, DUT...)
- ◯ Licence, licence pro, maîtrise, master 1
- ◯ Master, DEA, DESS, diplôme de grande école de niveau bac + 5 ou équivalent
- ◯ Doctorat de santé (médecine, pharmacie, dentaire)
- ◯ Doctorat hors santé

Page Break

Display This Question:

If Quel est le diplôme le plus élevé que vous ayez obtenu ? != Aucun diplôme

Q19 Êtes-vous un(e) professionnel(le) de santé ?

- Oui
- Non

Page Break

Q3 Combien d’autres personnes partagent le même logement que vous pendant cette période de confinement ?

- ○ Personne d'autre
- ○ 1
- ○ 2
- ○ 3
- ○ 4
- ○ 5
- ○ 6
- ○ 7
- ○ 8
- ○ 9
- ○ 10 ou plus

Page Break

Q43
Avant le confinement, vous a-t-on diagnostiqué une ou plusieurs des maladies suivantes

- ☐ ⊗ Non
- ☐ Sclérose en plaques
- ☐ Maladie auto-immune (psoriasis, maladie de Crohn, spondylarthrite ankylosante...)
- ☐ Maladie cardiovasculaire (insuffisance cardiaque...)
- ☐ Asthme
- ☐ Hypertension
- ☐ Diabète
- ☐ Cancer
- ☐ Broncho Pneumathie Chronique Obstructive (BPCO)
- ☐ Obésité

Page Break

Q92 Avez-vous le statut ALD (Affection Longue Durée) ?

- ○ Oui
- ○ Non
- ○ Je ne sais pas

Page Break

Display This Question:

If Combien d’autres personnes partagent le même logement que vous pendant cette période de confineme... != Personne d'autre

Q4 Partagez-vous votre domicile avec une personne vulnérable (personne âgée, handicapée, ou une personne atteinte d’une des maladies chroniques abordées à la question précédente) ?

- ▢ Oui, une personne âgée
- ▢ Oui, un enfant handicapé
- ▢ Oui, un enfant poly-handicapé
- ▢ Oui, un adulte handicapé
- ▢ Oui, un adulte poly-handicapé
- ▢ Oui, une personne atteinte d’une malade chronique
- ▢ ⊗Non

Page Break

Display This Question:

If Partagez-vous votre domicile avec une personne vulnérable (personne âgée, handicapée, ou une pers... != Non

And Combien d’autres personnes partagent le même logement que vous pendant cette période de confineme... != Personne d'autre

Q6 Si oui, est-ce que :

- ○ C’était déjà le cas avant le confinement
- ○ Cette personne est venue vivre avec vous pendant le confinement
- ○ Vous vous êtes installé(e) chez elle pendant le confinement

Page Break

Q93 Prenez-vous soin d’une personne vulnérable (âgée, handicapée ou atteinte d’une maladie chronique), avec qui vous ne vivez pas ?

- ☐ ⊗ Non
- ☐ Oui, une personne âgée
- ☐ Oui, un enfant handicapé
- ☐ Oui, un adulte handicapé
- ☐ Oui, une personne atteinte d’une maladie chronique

Page Break

Display This Question:

If Prenez-vous soin d’une personne vulnérable (âgée, handicapée ou atteinte d’une maladie chronique)... != Non

Q94 Preniez-vous déjà soin d'elle avant le confinement ?

- Oui
- Non

Page Break

Q14 Êtes-vous dans votre résidence principale (locataire ou propriétaire) ?

- ○ Oui
- ○ Non, je suis chez un membre de ma famille
- ○ Non, je suis dans ma résidence secondaire
- ○ Autre

Page Break

Q11 Combien de pièces d'habitation contient le logement dans lequel vous êtes confiné(e) ?

- ○ 1
- ○ 2
- ○ 3
- ○ 4
- ○ 5
- ○ Plus de 5

Page Break

Q12 Avez-vous un accès direct à un environnement extérieur (balcon, terrasse, jardin, cour... ?)

- Oui
- Non

Page Break

Display This Question:

If Combien d’autres personnes partagent le même logement que vous pendant cette période de confineme... != Personne d'autre

Q97 Combien d’enfants scolarisés sont confiné(s) avec vous ?

○ 0

○ 1

○ 2

○ 3

○ 4

○ 5 ou plus

Page Break

Display This Question:

If Combien d’enfants scolarisés sont confiné(s) avec vous ? != 0

And Combien d’autres personnes partagent le même logement que vous pendant cette période de confineme... != Personne d'autre

Q16 Less enfants confinés avec vous ont-ils des difficultés pour participer à l'enseignement à distance ?

- ▢ Oui, ils ont du mal à se concentrer
- ▢ Oui, je n’ai pas le temps de les aider
- ▢ Oui, nous n'avons pas de matériel informatique
- ▢ Oui, car il n’y a pas assez d'ordinateurs pour les personnes qui doivent travailler (enseignement à distance, télétravail)
- ▢ Oui, car nous n'avons pas accès à internet ou nous avons des problèmes pour nous connecter
- ▢ L'établissement scolaire n'a pas mis en place d'enseignement à distance
- ▢ ⊗Non applicable
- ▢ ⊗Non

Page Break

Display This Question:

If Combien d’autres personnes partagent le même logement que vous pendant cette période de confineme... != Personne d'autre

Q100
Combien d’enfants qui ne sont pas encore en âge d'être scolarisés sont confinés avec vous ?

- 0
- 1
- 2
- 3
- 4
- 5 ou plus

Page Break

Display This Question:

If Quelle est votre situation professionnelle ? != Retraité(e)

And Quelle est votre situation professionnelle ? != Autre

And Quelle est votre situation professionnelle ? != Étudiant(e)

Q18 Quel impact a l'épidémie de coronavirus sur votre emploi ?

- ▢ Je télétravaille à temps plein
- ▢ Je télétravaille à temps partiel
- ▢ Je continue d'aller travailler
- ▢ Je suis en chômage partiel
- ▢ Je suis en arrêt maladie
- ▢ Je suis en arrêt pour garder les enfants
- ▢ Je ne peux plus travailler car mon lieu de travail est fermé pendant le confinement (restaurant, bar, salon de coiffure…)
- ▢ Je ne peux plus travailler car je n'ai plus de clients en ce moment
- ▢ Mon chiffre d'affaire est nul pendant le confinement
- ▢ ⊗Aucun

Page Break

Display This Question:

If Combien d’enfants scolarisés sont confiné(s) avec vous ? != 0

Or Combien d’enfants qui ne sont pas encore en âge d'être scolarisés sont confinés avec vous ? != 0

And If

Quel impact a l’épidémie de coronavirus sur votre emploi ? = Je télétravaille à temps plein

Or Quel impact a l’épidémie de coronavirus sur votre emploi ? = Je télétravaille à temps partiel

And If

Combien d’autres personnes partagent le même logement que vous pendant cette période de confineme... != Personne d'autre

Q20 Quelle est votre organisation pour télétravailler et vous occuper des enfants ?

- ☐ Je télétravaille à temps partiel pour m'occuper d'eux
- ☐ Mon (ma) partenaire s'occupe d'eux
- ☐ Je m’en occupe moi-même
- ☐ Un autre membre du foyer s'en occupe
- ☐ Autre

Page Break

Display This Question:

If Combien d’enfants scolarisés sont confiné(s) avec vous ? != 0

Or Combien d’enfants qui ne sont pas encore en âge d'être scolarisés sont confinés avec vous ? != 0

And If

Quel impact a l’épidémie de coronavirus sur votre emploi ? = Je télétravaille à temps plein

Or Quel impact a l’épidémie de coronavirus sur votre emploi ? = Je télétravaille à temps partiel

And If

Combien d’autres personnes partagent le même logement que vous pendant cette période de confineme... != Personne d'autre

Q21 Le fait d'avoir des enfants à la maison pendant que vous travaillez a-t-il un impact sur la qualité de votre travail ?

- ☐ Oui, je travaille moins
- ☐ Oui, j’ai plus de mal à me concentrer
- ☐ ⊗ Pas du tout

Page Break

Q22 Avez-vous été diagnostiqué(e) positif au COVID-19 par un médecin ?

- ○ Oui
- ○ Non

Page Break

Display This Question:

If Avez-vous été diagnostiqué(e) positif au COVID-19 par un médecin ? = Oui

Q101 Le diagnostic a-t-il été confirmé par un test ?

- ☐ Oui, par prélèvement nasal
- ☐ Oui, par une prise de sang
- ☐ Oui, par un scanner
- ☐ ⊗Non

Page Break

Display This Question:

If Avez-vous été diagnostiqué(e) positif au COVID-19 par un médecin ? = Oui

Q23 Avez-vous été en contact (à moins de 2 mètres) d'un cas confirmé ?

- Oui
- Non
- Je ne sais pas

Page Break

Display This Question:

If Avez-vous été diagnostiqué(e) positif au COVID-19 par un médecin ? = Oui

Q24 Parmi les symptômes suivants, lesquels avez-vous éprouvés :

- ☐ Une toux ou une augmentation de votre toux habituelle
- ☐ Une forte diminution ou perte de votre goût ou de votre odorat
- ☐ Un mal de gorge et/ou des douleurs musculaires et/ou des courbatures inhabituelles
- ☐ Une fatigue inhabituelle ou qui vous oblige à vous reposer plus de la moitié de la journée
- ☐ Une impossibilité de vous alimenter ou de boire (dégoût quand vous voyez de la nourriture)
- ☐ Un manque de souffle inhabituel lorsque vous parlez ou faites un petit effort
- ☐ De la fièvre
- ☐ Une diarrhée
- ☐ Autre (précisez :) ____
- ☐ ⊗Aucun symptôme

Page Break

Display This Question:

If Avez-vous été diagnostiqué(e) positif au COVID-19 par un médecin ? != Oui

Q26 Pensez-vous avoir été contaminé(e) par le COVID 19 mais sans avoir de diagnostic confirmé ?

- Oui
- Non
- Je ne sais pas

Page Break

Display This Question:

If Avez-vous été diagnostiqué(e) positif au COVID-19 par un médecin ? != Oui

And Pensez-vous avoir été contaminé(e) par le COVID 19 mais sans avoir de diagnostic confirmé ? = Oui

Q27 Avez-vous éprouvé les symptômes suivants :

- ☐ Une toux ou une augmentation de votre toux habituelle
- ☐ Une forte diminution ou perte de votre goût ou de votre odorat
- ☐ Un mal de gorge et/ou des douleurs musculaires et/ou des courbatures inhabituelles
- ☐ Une fatigue inhabituelle ou qui vous oblige à vous reposer plus de la moitié de la journée
- ☐ Une impossibilité de vous alimenter ou de boire (dégoût quand vous voyez de la nourriture)
- ☐ Un manque de souffle inhabituel lorsque vous parlez ou faites un petit effort
- ☐ De la fièvre
- ☐ Une diarrhée
- ☐ Autre (précisez :) ____
- ☐ ⊗Aucun symptôme

Page Break

Q29 Connaissez-vous quelqu'un qui a été diagnostiqué positif au COVID-19 ?

- Oui
- Non
- Je ne sais pas

Page Break

Q102 L'un de vos proches est-il décédé du COVID-19 ?

- [ ] Oui, un proche vivant en EHPAD (maison de retraite)
- [ ] Oui, un proche qui ne vivait pas en EHPAD
- [x] Non

Page Break

Display This Question:

If Avez-vous été diagnostiqué(e) positif au COVID-19 par un médecin ? != Oui

Q30 Pensez-vous que si vous êtes contaminé(e) par le virus du COVID-19, vous risquez de développer une forme grave de la maladie ?

- Oui
- Non
- Je ne sais pas

Page Break

Display This Question:

If Pensez-vous que si vous êtes contaminé(e) par le virus du COVID-19, vous risquez de développer un... = Oui

Q31 Pourquoi ?

- ☐ Je fais partie des personnes fragiles les plus à risque
- ☐ Je sais que n'importe qui peut développer une forme grave de cette maladie
- ☐ Autre
- ☐ ⊗Je ne sais pas

Page Break

Display This Question:

If Pensez-vous que si vous êtes contaminé(e) par le virus du COVID-19, vous risquez de développer un... = Non

Q32 Pourquoi pensez-vous cela ?

- [ ] Je me dis que je suis suffisamment en bonne santé pour y résister
- [ ] Je prends des produits en prévention (homéopathie, vitamines, compléments alimentaires…)
- [ ] Je suis trop jeune pour développer une forme grave du COVID-19
- [ ] Je fais du sport et je m'entretiens régulièrement
- [ ] ⊗Je ne sais pas

Page Break

Display This Question:

If Avez-vous été diagnostiqué(e) positif au COVID-19 par un médecin ? = Non

Q45 Si vous pensez avoir des symptômes de COVID-19, vers qui vous tournerez-vous en premier ?

- ○ Je me rendrai au cabinet de mon médecin
- ○ Je demanderai à un médecin de venir chez moi
- ○ Téléconsultation avec mon médecin généraliste
- ○ Téléconsultation avec n'importe quel médecin
- ○ Urgences
- ○ Pharmacie
- ○ Un centre spécialisé pour le COVID
- ○ Autre (précisez :) ____

Page Break

Display This Question:

If Avez-vous été diagnostiqué(e) positif au COVID-19 par un médecin ? = Non

Q33 Pensez-vous que si vous êtes contaminé(e) par le virus du COVID-19, vous mettrez en danger la santé d’un de vos proches ?

- Oui
- Non
- Je ne sais pas

Page Break

Display This Question:

If Combien d’autres personnes partagent le même logement que vous pendant cette période de confineme... != Personne d'autre

Q103 Pensez-vous qu’un des membres de votre logement pourrait développer une forme grave, en cas de contamination par le virus du COVID-19 ?

- Oui
- Non
- Je ne sais pas

Page Break

Q104 Pensez-vous qu'en restant chez vous et en vous isolant, vous avez un impact sur la pandémie ?

- ○ Oui
- ○ Non

Page Break

Q34 Respectez-vous le confinement ?

- Oui, je respecte les règles très sérieusement
- Oui, j’essaye de ne pas trop déroger aux règles
- Non, je ne pense pas
- Non, je trouve ce confinement exagéré

Page Break

Q35 Sur une échelle de 1 à 10 (1 étant le plus bas niveau d'inquiétude et 10 le plus haut), comment estimez-vous votre niveau d'inquiétude :

	1	2	3	4	5	6	7	8	9	10
concernant l'impact du COVID-19 sur votre santé personnelle										
concernant l'impact du COVID-19 pour vos proches										
concernant l'impact de la crise sur vos finances personnelles										

Page Break

Q36 Votre inquiétude a-t-elle évolué ces deux dernières semaines ?

- Oui, elle a augmenté
- Oui, elle a diminué
- Non

Page Break

Q48 L’un de vos proches est-il en EHPAD (maison de retraite) en ce moment ?

- ○ Oui
- ○ Non

Page Break

Q39 Le confinement a-t-il un impact sur votre libido ?

- Augmentation du désir
- Diminution du désir
- Non

Page Break

Q40 Prenez-vous régulièrement des médicaments ?

- ○ Oui
- ○ Non

Page Break

Display This Question:

If Prenez-vous régulièrement des médicaments ? = Oui

Q41 Vos médicaments sont-ils encore disponibles ou en rupture de stock ?

- Encore disponibles
- En rupture de stock
- Je ne sais pas

Page Break

Display This Question:

If Avez-vous été diagnostiqué(e) positif au COVID-19 par un médecin ? = Oui

And Prenez-vous régulièrement des médicaments ? = Oui

Q42 Avez-vous dû interrompre votre traitement ou votre médecin envisage-t-il de le faire pendant la pandémie ?

- ○ Oui
- ○ Non
- ○ Je ne sais pas

Page Break

Q105 Avant le confinement, receviez-vous régulièrement des soins ?

- ○ Oui
- ○ Non

Page Break

Q44 Quel est l'impact de la pandémie sur l'organisation de votre suivi médical ?

- ☐ Mon ou mes rendez-vous de dépistage (mammographie, coloscopie, examen dermatologique) ont été annulés ou reportés
- ☐ Mes autres rendez-vous médicaux ont été annulés ou reportés
- ☐ Mes rendez-vous médicaux ont été maintenus
- ☐ J'ai moi-même décidé de reporter mes soins ou examens médicaux, je pense que cela peut attendre
- ☐ J'ai moi-même décidé de reporter mon ou mes examens de dépistage, je pense que cela peut attendre
- ☐ Je ne peux pas me rendre à mon rendez-vous parce que le cabinet du médecin est fermé
- ☐ Je ne peux pas me rendre à mon rendez-vous car il n'y a plus assez de transports en commun
- ☐ Mon opération a été reportée ou annulée
- ☐ Les médicaments dont j'ai besoin sont en rupture de stock
- ☐ Je ne reçois plus de soins à domicile
- ☐ Je reçois moins de soins à domicile
- ☐ ⊗Aucun impact

Page Break

Display This Question:

If Combien d’enfants scolarisés sont confiné(s) avec vous ? != 0

Or Combien d’enfants qui ne sont pas encore en âge d'être scolarisés sont confinés avec vous ? != 0

And If

Combien d’autres personnes partagent le même logement que vous pendant cette période de confineme... != Personne d'autre

Q106 Quel est l’impact de la pandémie sur le suivi médical de votre(vos) enfant(s) ?

- ☐ Les rendez-vous médicaux pour mes enfants ont été annulés ou décalés
- ☐ J’ai moi-même décidé de reporter les rendez-vous médicaux pour mes enfants, je pense que cela peut attendre
- ☐ Un rendez-vous pour faire les vaccins a été annulé ou décalé
- ☐ J’ai annulé ou décalé moi-même un rendez-vous pour faire les vaccins, je pense que cela peut attendre
- ☐ ⊗Il n'y avait pas de vaccin à faire pendant le confinement
- ☐ Les médicaments dont il a (ils ont) besoin sont en rupture de stock
- ☐ ⊗Aucun impact

Page Break

Q46 Avez-vous fait une téléconsultation avec un professionnel de santé depuis le début du confinement ?

- [ ] Oui, pour moi
- [ ] Oui, pour un des enfants
- [ ] ⊗ Non

Page Break

Display This Question:

If Avez-vous fait une téléconsultation avec un professionnel de santé depuis le début du confinement... != Non

Q114 Avez-vous fait une téléconsultation :

- ☐ Avec votre médecin traitant ou le médecin traitant d'un des enfants
- ☐ Avec un spécialiste qui vous suit habituellement ou qui suit habituellement les enfants
- ☐ Avec un médecin généraliste que vous ou votre enfant n’avez jamais consulté auparavant
- ☐ Avec un médecin spécialiste que vous ou votre enfant n’avez jamais consulté auparavant

Page Break

Display This Question:

If Avez-vous fait une téléconsultation avec un professionnel de santé depuis le début du confinement... = Oui, pour moi

Or Avez-vous fait une téléconsultation avec un professionnel de santé depuis le début du confinement... = Oui, pour un des enfants

Q107 Avec quelles propositions suivantes concernant la téléconsultation êtes-vous d’accord ?

- ▢ Je n’apprécie pas de parler avec un médecin à distance
- ▢ J’ai peur pour mes données personnelles
- ▢ Je trouve cela pratique
- ▢ Je souhaite continuer d'utiliser la téléconsultation après le confinement
- ▢ Mon médecin traitant n’est pas équipé

Page Break

Display This Question:

If Avez-vous fait une téléconsultation avec un professionnel de santé depuis le début du confinement... = Non

Q108 Pourquoi n'avez-vous pas fait de téléconsultation depuis le début du confinement ?

- ▢ Je ne sais pas comment faire
- ▢ Je n'ai pas envie de parler à un médecin à distance
- ▢ J'ai peur pour mes données personnelles
- ▢ Mon médecin n'est pas équipé
- ▢ Je n'en ai pas eu besoin mais j'en ferai une si nécessaire
- ▢ Je n'en ai pas eu besoin et je n'en ferai pas si je dois consulter

---

Page Break

Q49 Avez-vous besoin d'une aide régulière à votre domicile par un professionnel (auxiliaire de vie, soins à domicile, repas à domicile...) pour vous ou l'un des membres de votre foyer ? (si oui, veuillez préciser quel type de professionnel)

- [ ] Oui, pour moi ____
- [ ] Oui, pour un autre membre de mon foyer ____
- [ ] ⊗ Non

Page Break

Display This Question:

If Avez-vous besoin d'une aide régulière à votre domicile par un professionnel (auxiliaire de vie, s... = Oui, pour moi

Or Avez-vous besoin d'une aide régulière à votre domicile par un professionnel (auxiliaire de vie, s... = Oui, pour un autre membre de mon foyer

Q50 Ces services et l'assistance de ces professionnels étaient-ils déjà en place avant le COVID ?

- ○ Oui
- ○ Non

Page Break

Display This Question:

If Avez-vous besoin d'une aide régulière à votre domicile par un professionnel (auxiliaire de vie, s... = Oui, pour moi

Or Avez-vous besoin d'une aide régulière à votre domicile par un professionnel (auxiliaire de vie, s... = Oui, pour un autre membre de mon foyer

And If

Ces services et l'assistance de ces professionnels étaient-ils déjà en place avant le COVID ? = Oui

Q51 La venue de ce(s) professionnel(s) est-elle impactée par la pandémie ?

- ☐ Oui, il y a moins de passages que nécessaire
- ☐ Oui, certains professionnels ne viennent plus du tout
- ☐ Oui, plus aucun professionnel ne vient
- ☐ ⊗Non

Page Break

Display This Question:

If Avez-vous besoin d'une aide régulière à votre domicile par un professionnel (auxiliaire de vie, s... = Oui, pour moi

Or Avez-vous besoin d'une aide régulière à votre domicile par un professionnel (auxiliaire de vie, s... = Oui, pour un autre membre de mon foyer

And If

Ces services et l'assistance de ces professionnels étaient-ils déjà en place avant le COVID ? = Non

Q115 La venue de ce(s) professionnel(s) est-elle impactée par la pandémie ?

- [ ] Oui, il y a moins de passages que nécessaire
- [ ] Oui, certains professionnels ne viennent pas du tout
- [ ] Oui, aucun professionnel ne vient
- [ ] ⊗Non

Page Break

Display This Question:

If Partagez-vous votre domicile avec une personne vulnérable (personne âgée, handicapée, ou une pers... = Oui, une personne âgée

Or Partagez-vous votre domicile avec une personne vulnérable (personne âgée, handicapée, ou une pers... = Oui, un enfant handicapé

Or Partagez-vous votre domicile avec une personne vulnérable (personne âgée, handicapée, ou une pers... = Oui, un enfant poly-handicapé

Or Partagez-vous votre domicile avec une personne vulnérable (personne âgée, handicapée, ou une pers... = Oui, un adulte handicapé

Or Partagez-vous votre domicile avec une personne vulnérable (personne âgée, handicapée, ou une pers... = Oui, un adulte poly-handicapé

Or Partagez-vous votre domicile avec une personne vulnérable (personne âgée, handicapée, ou une pers... = Oui, une personne atteinte d'une malade chronique

Or Prenez-vous soin d'une personne vulnérable (âgée, handicapée ou atteinte d'une maladie chronique)... = Oui, une personne âgée

Or Prenez-vous soin d'une personne vulnérable (âgée, handicapée ou atteinte d'une maladie chronique)... = Oui, un enfant handicapé

Or Prenez-vous soin d'une personne vulnérable (âgée, handicapée ou atteinte d'une maladie chronique)... = Oui, un adulte handicapé

Or Prenez-vous soin d'une personne vulnérable (âgée, handicapée ou atteinte d'une maladie chronique)... = Oui, une personne atteinte d'une maladie chronique

Q52 Effectuez-vous partiellement ou totalement les soins d'une personne âgée, handicapée ou atteinte d'une malade chronique ?

- ☐ Oui, sa toilette
- ☐ Oui, la préparation des médicaments
- ☐ Autre (précisez :) ____
- ☐ ⊗Non

Page Break

Display This Question:

If Effectuez-vous partiellement ou totalement les soins d'une personne âgée, handicapée ou atteinte... = Oui, sa toilette

Or Effectuez-vous partiellement ou totalement les soins d'une personne âgée, handicapée ou atteinte... = Oui, la préparation des médicaments

Or Effectuez-vous partiellement ou totalement les soins d'une personne âgée, handicapée ou atteinte... = Autre (précisez :)

Q53 Avec quelles propositions ci-dessous êtes-vous d'accord ?

- ▢ Je le faisais déjà habituellement avant le confinement
- ▢ Je le faisais déjà habituellement avant le confinement, mais ce n'est pas par choix mais par manque d'aide extérieure
- ▢ Je le fais par choix, je ne veux pas que des professionnels viennent au domicile et le(la) contaminent
- ▢ Je n'ai pas le choix, il n'y a plus assez de professionnels pour s'en occuper
- ▢ Je n'ai pas le choix, aucune aide à domicile n'est mise en place
- ▢ C'est normal à mes yeux de réaliser moi-même ces tâches
- ▢ C'est à un professionnel de réaliser ces tâches, pas à moi

Page Break

Q54 Avez-vous changé la fréquence de vos lavages des mains depuis le début de la pandémie ?

- Oui, je me les lave plus souvent
- Oui je me les lave moins souvent
- Non

Page Break

Q55 Combien de temps vous lavez-vous les mains en moyenne ?

- 5 - 10 secondes
- 10 - 20 secondes
- Plus de 20 secondes

Page Break

Q56 Depuis le début du confinement, avez-vous changé vos habitudes alimentaires ?

- ○ Oui, je mange plus équilibré
- ○ Oui je mange moins équilibré
- ○ Non

Page Break

Display This Question:

If Depuis le début du confinement, avez-vous changé vos habitudes alimentaires ? = Oui je mange moins équilibré

Or Depuis le début du confinement, avez-vous changé vos habitudes alimentaires ? = Oui, je mange plus équilibré

Q58 Par rapport à avant le confinement, qu’avez-vous changé dans votre alimentation ?

- ☐ Je mange plus de fruits et légumes
- ☐ Je mange moins de fruits et légumes
- ☐ J’ai moins d’appétit parce que je suis stressé(e) ou triste
- ☐ J’ai plus d’appétit parce que je suis stressé(e) ou triste
- ☐ Je fais la cuisine plus souvent parce que j'ai plus de temps
- ☐ J'ai moins le temps de cuisiner
- ☐ Je ne sais pas comment cuisiner équilibré
- ☐ Je ne suis pas motivé(e) pour cuisiner par moi-même
- ☐ Un de mes proches cuisine pour moi pendant le confinement
- ☐ Je commande moins de plats à emporter
- ☐ Je commande plus de plats à emporter
- ☐ J’utilise moins les services de livraison de repas à domicile
- ☐ J’utilise plus souvent les services de livraison de repas à domicile
- ☐ Je trouve plus difficile d'acheter des produits sains
- ☐ Le prix des produits sains a augmenté et mon budget ne me le permet plus
- ☐ Je grignote plus pendant mes journées de travail
- ☐ Je grignote moins pendant que je travaille
- ☐ Je grignote souvent en regardant la télévision ou en surfant sur internet

Page Break

Q59 Depuis le début du confinement, la durée de votre sommeil a-t-elle changé ?

- ○ Oui, je dors plus longtemps
- ○ Oui, je dors moins longtemps
- ○ Non

Page Break

Q109 Depuis le début du confinement, au réveil :

- ○ Je me sens souvent reposé(e)
- ○ Je ne me sens pas souvent reposé(e)

Page Break

Q110 Avec quelles propositions concernant votre sommeil êtes-vous d’accord ?

- ☐ J'ai l'impression de trop dormir depuis le début du confirment
- ☐ J’ai l’impression de ne pas dormir assez depuis le début du confirment
- ☐ Je suis fatigué(e) pendant la journée depuis le début du confirment
- ☐ J’ai l’impression de mieux dormir qu’avant le confinement
- ☐ J’ai l’impression de moins bien dormir qu’avant le confinement
- ☐ Je ne vois pas de changement dans ma qualité de sommeil depuis le début du confinement

Page Break

Q61 Depuis le début du confinement, diriez-vous que :

- ☐ Vous avez pris l'habitude de faire une ou plusieurs siestes longues
- ☐ Votre horaire de coucher varie tous les jours
- ☐ Vous vous réveillez plus souvent dans la nuit
- ☐ Vous vous réveillez moins souvent dans la nuit
- ☐ Vous faites plus de cauchemars qu'avant
- ☐ Vous faites moins de cauchemars qu'avant
- ☐ Votre horaire de lever varie tous les jours (et c'était déjà le cas avant le confinement)
- ☐ Votre horaire de lever varie tous les jours (et ça a commencé pendant le confinement)
- ☐ Vos horaires de travail sont plus étendus, vous devez vous lever plus tôt ou vous coucher plus tard
- ☐ Vous prenez des somnifères pour réussir à dormir
- ☐ Vous n'arrivez pas à vous détendre avant de vous coucher
- ☐ Vous avez plus de mal à vous endormir qu'avant
- ☐ ⊗Aucune de ces réponses

Page Break

Display This Question:

If Depuis le début du confinement, diriez-vous que : = Vous avez pris l'habitude de faire une ou plusieurs siestes longues

Q84 Comment caractériseriez-vous vos siestes ?

- ☐ Moins de 30 min
- ☐ 30 min à 1h
- ☐ Plus d'une heure
- ☐ Dans mon lit
- ☐ Dans un fauteuil ou canapé
- ☐ Je m'endors devant la télé
- ☐ Je m'endors en lisant
- ☐ Je m'endors en étant allongé(e)
- ☐ Je m'endors assis(e)

Page Break

Q62 Quel impact a le confinement sur votre activité physique ?

- ○ J’ai commencé à faire de l'activité physique
- ○ Je fais plus d'activité physique
- ○ Je fais moins d'activité physique
- ○ J'ai arrêté de faire de l'activité physique
- ○ Pas de changement

Page Break

Display This Question:

If Quel impact a le confinement sur votre activité physique ? = J'ai commencé à faire de l'activité physique

Or Quel impact a le confinement sur votre activité physique ? = Je fais plus d'activité physique

Q63 Comment pratiquez-vous ?

- ☐ Je fais du sport en intérieur sans équipement lourd (vélo d'appartement, tapis de course…)
- ☐ Je fais du sport en intérieur avec un équipement lourd (vélo d'appartement, tapis de course…)
- ☐ Je sors faire des promenades régulièrement
- ☐ Je sors faire de l'activité physique
- ☐ Je sors faire du jogging régulièrement
- ☐ Autre

Page Break

Display This Question:

If Quel impact a le confinement sur votre activité physique ? = Je fais moins d'activité physique

Or Quel impact a le confinement sur votre activité physique ? = J'ai arrêté de faire de l'activité physique

Q64 Pour quelle(s) raison(s) faites-vous moins d'activité physique ou l'avez-vous complètement arrêtée ?

- [ ] Je n'ai pas le temps de faire de l'exercice
- [ ] Je ne sors plus et ne fais pas de sport chez moi
- [ ] Je n'ai pas la place pour faire de l'exercice
- [ ] Je ne sais pas quel exercice faire à la maison
- [ ] Je n'ai pas l'équipement nécessaire pour faire de l'exercice
- [ ] Je ne suis pas motivé(e) pour faire de l'exercice seul(e)
- [ ] Je suis trop fatigué(e) pour faire de l'exercice
- [ ] Je n’ai plus envie de faire de l’exercice
- [ ] Je suis trop malade pour faire de l'exercice
- [ ] Autre

Page Break

Q65 Depuis le début du confinement, avez-vous changé vos habitudes de consommation d'alcool ?

- Oui, je bois plus
- Oui, je bois moins
- Non, pas de changement
- Je ne bois pas d'alcool

Page Break

Display This Question:

If Depuis le début du confinement, avez-vous changé vos habitudes de consommation d'alcool ? = Oui, je bois plus

Q66 Pourquoi buvez-vous plus ?

- ☐ L'alcool m’aide à surmonter mon ennui
- ☐ L’alcool m’aide à surmonter mon anxiété
- ☐ L’alcool m’aide à surmonter ma tristesse
- ☐ L’alcool m’aide à surmonter ma solitude
- ☐ Je me récompense avec un verre après une longue journée de travail
- ☐ J'apprécie un verre après une journée stressante avec les enfants
- ☐ Je veux rester virtuellement en contact avec mes amis (par exemple « apéro-virtuel »)
- ☐ Boire régulièrement est devenu une habitude sans que je m'en rende compte
- ☐ Autre

Page Break

Display This Question:

If Depuis le début du confinement, avez-vous changé vos habitudes de consommation d'alcool ? = Oui, je bois moins

Q67 Pourquoi avez-vous diminué votre consommation d’alcool ?

- ▢ Je n'ai actuellement pas les moyens d'acheter de l'alcool
- ▢ Je ne bois pas d'alcool parce que j'ai peur d'attraper le COVID-19 dans le magasin
- ▢ D’habitude, je ne bois que lorsque je sors
- ▢ D’habitude, je ne bois qu'avec mes amis, mes collègues ou ma famille
- ▢ Autre

Page Break

Display This Question:

If Depuis le début du confinement, avez-vous changé vos habitudes de consommation d'alcool ? != Je ne bois pas d'alcool

Q111 Quelle est votre consommation d’alcool depuis le début du confinement ?

- ▢ Je bois 3 verres ou plus chaque jour
- ▢ Je bois tous les jours
- ▢ Je bois plus de 10 verres par semaine
- ▢ Je bois moins de 3 verres par jour
- ▢ Je passe des journées sans boire
- ▢ Je bois moins de 10 verres par semaine

Page Break

Q68 Depuis le début du confinement, avez-vous modifié votre consommation de tabac ?

- ○ Oui, je fume plus
- ○ Oui, je fume moins
- ○ Non, je fume toujours autant
- ○ Je ne fume pas

Page Break

Display This Question:

If Depuis le début du confinement, avez-vous modifié votre consommation de tabac ? = Oui, je fume plus

Q69 Pourquoi fumez-vous plus ?

- ☐ Fumer m'aide à surmonter mon ennui
- ☐ Fumer m'aide à déstresser
- ☐ En télétravaillant, j'ai davantage l'occasion de fumer
- ☐ Je n’y pense pas, c’est un réflexe
- ☐ Autre

Page Break

Display This Question:

If Depuis le début du confinement, avez-vous modifié votre consommation de tabac ? = Oui, je fume moins

Q70 Pourquoi avez-vous diminué votre consommation de tabac ?

- ▢ J’ai du mal à trouver un bureau de tabac ouvert près de chez moi
- ▢ J'ai peur d'être contaminé(e) dans le bureau de tabac
- ▢ Je n'ai actuellement pas les moyens d'acheter du tabac
- ▢ Je n'ai pas la possibilité de fumer quand je suis à la maison avec les enfants
- ▢ Je veux réduire mes risques de développer une forme grave du COVID-19
- ▢ Autre

Page Break

Q71 Depuis le début du confinement, avez-vous augmenté votre temps d'écran ?

- ☐ Oui, pour le travail
- ☐ Oui, pour les loisirs
- ☐ Oui, pour une autre raison
- ☐ ⊗ Non

Page Break

Q74 Au cours des 7 derniers jours, pourquoi êtes-vous sorti(e) ?

- [ ] ⊗Je ne sors pas
- [ ] Pour m'aérer
- [ ] Pour promener les enfants
- [ ] Pour promener mon animal domestique
- [ ] Pour faire mes courses
- [ ] Pour faire de l'exercice
- [ ] Pour rendre visite à des amis
- [ ] Pour m'occuper d'un proche vulnérable
- [ ] En raison de la garde alternée de mon (mes) enfant(s)
- [ ] Pour aller travailler
- [ ] Pour aller chez le médecin ou à la pharmacie
- [ ] Autre

Page Break

Q75 Comment décririez-vous votre vie sociale pendant le confinement ?

- ☐ Je reste en contact par téléphone ou par internet avec le reste de mes amis ou de ma famille
- ☐ Je n'ai pas de contact avec le monde extérieur parce que je le souhaite
- ☐ Je n'ai pas de contact avec le monde extérieur parce que personne ne me demande de mes nouvelles
- ☐ Je passe moins de temps avec mes amis mais je sors quand même pour leur rendre visite
- ☐ Je n’ai plus envie d’être en contact avec les autres
- ☐ ⊗ Aucune des propositions ci-dessus

Page Break

Q76 Diriez-vous que depuis le début du confinement :

- ☐ Vous passez plus de temps de qualité avec vos proches
- ☐ Vous aidez plus les autres (courses pour une personne vulnérable, fabrication de masques, bénévolat...)
- ☐ Vous regardez ou lisez plus les informations que d'habitude
- ☐ Vous ne supportez plus de regarder ou de lire les informations
- ☐ Vous vous sentez plus seul(e)
- ☐ Vous vous sentez plus déprimé(e)
- ☐ Vous vous sentez plus stressé(e)
- ☐ Vous vous sentez plus irritable
- ☐ ⊗Aucune des réponses précédentes

Page Break

Q116 Diriez-vous que depuis le début du confinement :

- ▢ Les relations avec votre partenaire sont plus tendues
- ▢ Vous vous sentez plus proche de votre partenaire
- ▢ Les relations avec les enfant(s) sont plus tendues
- ▢ Vous vous sentez plus proche des enfants
- ▢ Les relations avec un ou plusieurs autres membres de votre famille sont plus tendues
- ▢ Vous vous sentez plus proche d'un ou plusieurs autres membres de votre famille
- ▢ Une personne confinée avec vous est devenue plus agressive
- ▢ ⊗Aucune des réponses précédentes

Page Break

Q77 Avez-vous dû annuler ou reporter l'un de ces évènements en raison de la pandémie ?

- ☐ Mon mariage
- ☐ Participation à un mariage
- ☐ Participation à des funérailles
- ☐ Mon anniversaire
- ☐ Participation à un anniversaire
- ☐ Vacances
- ☐ Rassemblement religieux
- ☐ Examen universitaire
- ☐ Autre
- ☐ ⊗Non, aucun évènement

Page Break

Q78 Quel est le risque que vous rencontriez des difficultés financières dans les prochains mois à cause du confinement et de la pandémie ?

- ○ Risque très élevé
- ○ Risque plutôt élevé
- ○ Risque plutôt faible
- ○ Aucun risque

Page Break

Q79 La pandémie a-t-elle un impact sur vos revenus ?

- ○ Oui, ils ont diminué
- ○ Oui, ils ont augmenté
- ○ Non, pas de changement

End of Block: Default Question Block
